# Tissue sampling for biliary strictures using novel elbow biopsy forceps

**DOI:** 10.1038/s41598-021-90197-4

**Published:** 2021-05-25

**Authors:** Huahui Zhang, Chunyan Huo, Yongxin Guo, Keyuan Zhu, Fengdong Li, Jin Huang

**Affiliations:** 1 Graduate School of Dalian Medical University, Dalian, China; 2 Department of Gastroenterology, Changzhou Second People’s Hospital Affiliated to Nanjing Medical University, Changzhou, China

**Keywords:** Gastroenterology, Biliary tract disease

## Abstract

We aim to evaluate the safety and feasibility of novel elbow biopsy forceps with a prebent head for sampling biliary strictures in our institution. A total of 24 patients (15 males and 9 females) with biliary stricture who underwent biliary biopsy during endoscopic retrograde cholangiopancreatography (ERCP) using novel elbow biopsy forceps from June 2019 to August 2020 were retrospectively included. The novel biopsy forceps had a head angulation of 30 degrees and were able to cannulate the bile duct and approach the biliary strictures easily to obtain adequate samples. The technical success rate, incidence of adverse events, and consistency of pathological and surgical specimens were assessed. This device was used successfully in all patients. A total of 52 biopsy specimens were obtained from 24 patients, and all specimens could be used for histopathological examination. Seventeen patients were diagnosed with malignancy based on biopsies, and all of them underwent surgical treatment. The histopathological findings of the biopsy specimens were in accordance with the postoperative pathology diagnoses. One of the seven patients was diagnosed with a benign lesion that was proven to be malignant during surgical treatment in the follow-up period. Two patients experienced a single episode of acute pancreatitis and recovered shortly after appropriate treatment. No patients experienced biliary perforation or biliary bleeding. Biopsy using novel elbow forceps in patients with biliary stenosis is feasible and safe. The novel device and related biopsy technique may be widely applied for biliary disease differentiation.

## Introduction

Biliary stricture is narrowing of the biliary tree and is induced by various causes. Malignant biliary stricture is caused by pancreatic cancer, cholangiocarcinoma, ampullary cancer, and other malignant diseases^1^. Benign biliary strictures are induced by inflammatory diseases, choledocholithiasis, and postoperative adverse events^2^. Unfortunately, most biliary strictures are malignant and three to five times more prevalent than benign strictures^3^. Even imaging technology, which is constantly improving, is still unable to accurately differentiate between benign and malignant strictures^4,5^. Histopathological examination is the gold standard for the diagnosis of biliary tract tumors; therefore, it is particularly important to obtain adequate biopsy specimens from the bile duct.

Developing a new device enabling the quick diagnosis of indeterminate biliary strictures is important. Biliary tract biopsy with biopsy forceps or a biliary brush during ERCP is still the first choice due to the high cost of cholangioscopy. According to previous studies, the sensitivity resulting from the use of biopsy forceps is higher than that of the biliary brushing technique, as ERCP is easy to perform and associated with few adverse events^6,7^. However, the sensitivity resulting from the use of biopsy forceps ranges from 31 to 65%^8–10^. The reason for its low sensitivity is the inability to obtain adequate specimens. In addition, accessing the bile duct and maintaining close contact with the bile duct wall are difficult with conventional biopsy forceps, which have a straight head.

In this study, we introduced novel elbow biopsy forceps for biliary biopsy that are more flexible and easier to use for bile duct intubation. The main aim of this study was to evaluate the success rate and safety of this device and its related biopsy procedure.

## Methods

### Patients

**Figure 1:**
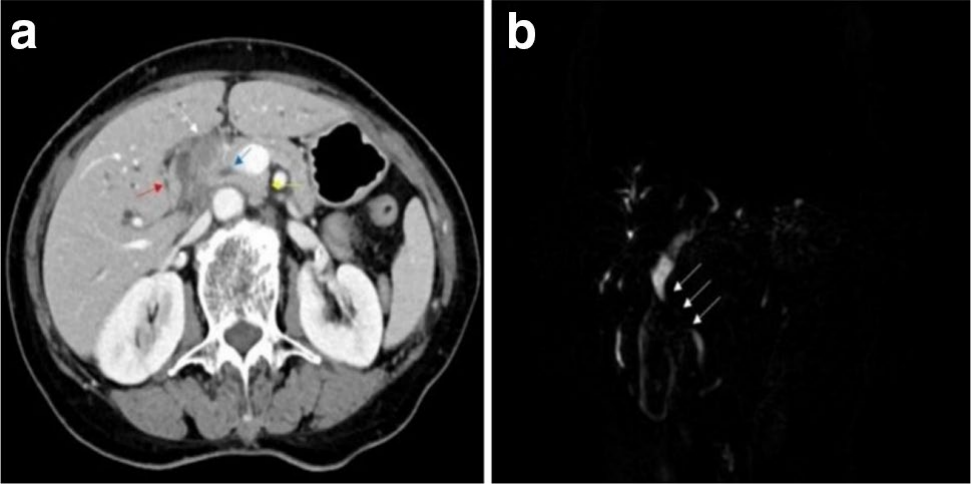
Computed tomography (**a**) and magnetic resonance imaging (**b**) showed stenosis at the common hepatic duct and the upper segment of the common bile duct (**a** The white arrow indicates the soft tissue shadow, the blue arrow indicates the common bile duct, the red arrow indicates the duodenum, and the yellow arrow indicates the head of the pancreas. **b** The white arrow indicates the stenosis.).

Patients with biliary stricture were recruited from June 2019 to August 2020 at Changzhou Second People's Hospital affiliated to Nanjing Medical University. The inclusion criteria for our study were as follows: (1) patients with biliary stricture confirmed by computed tomography and magnetic resonance imaging (Fig. 1); and (2) patients who had undergone biliary biopsy with novel elbow biopsy forceps. The exclusion criteria were as follows: (1) patients who had undergone biliary biopsy or brushing in the past; and (2) patients who had a history of malignant biliary tumors.

A total of 24 patients were enrolled in our study. None of the patients had contradictions for ERCP, and all patients gave written informed consent before the ERCP and biopsy procedures. The study was conducted in Changzhou Second People's Hospital affiliated to Nanjing Medical University in accordance with the guidelines of the National Institutes of Health of China. This study was approved by the Ethics Committee of Changzhou Second People's Hospital affiliated to Nanjing Medical University.

### Primary and secondary outcomes

The primary outcome measures of this study were the rate of technical success, the ERCP-related adverse event rate, and the forceps-related adverse event rate. The secondary outcome was the pathological coincidence rate. Technical success was defined as obtaining biopsy specimens that could be used for histopathological examination. ERCP-related adverse events included bleeding, perforation, pancreatitis, cholangitis, and even death. Forceps-related adverse events included biliary bleeding and perforation. Pathological coincidence was defined as consistent pathological and surgical specimen results. Regarding the histopathologic diagnosis of the specimens, neoplastic lesions and suspected malignancies were considered malignant, and the remaining findings were benign.

### Elbow biopsy forceps

**Figure 2:**
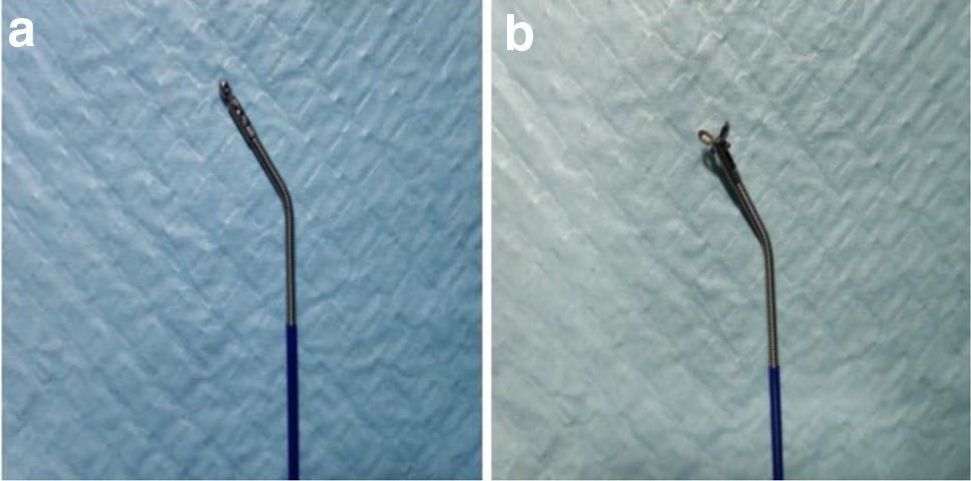
Novel elbow biopsy forceps with a head angulation of 30 degrees.

We developed novel elbow biopsy forceps with a head angulation of 30 degrees. Since the head of this elbow biopsy forceps is bent and ductile, bile duct intubation can be more easily performed (Supplementary Video 1). The novel biopsy forceps have a powerful occlusal force due to a four-bar linkage mechanism. Furthermore, the forceps have a wide clamp distance (7.1 mm), and the outer diameter of the forceps is 2 mm (Fig. 2). Second, the forceps can better approach the bile duct wall and obtain adequate specimens. Endoscopic sphincterotomy and papillary balloon dilation are also required before biliary intubation by elbow forceps. In addition, the forceps can be inserted into the bile duct without the guidance of a guide wire, which is different from conventional biopsy forceps.

### ERCP

ERCP was performed by gastroduodenal endoscopy. First, a gastroduodenal endoscope was advanced to the duodenal papilla. Next, bile duct cannulation was performed with a CleverCut sphincterotome. Then, the biliary stricture was shown by cholangiography with meglumine diatrizoate. Finally, endoscopic sphincterotomy and papillary balloon dilation of the papilla were performed to 3 mm and 8 mm, respectively. ERCP was completed under anesthesia and analgosedation in an endoscopic operating room.

### Biliary biopsy

**Figure 3:**
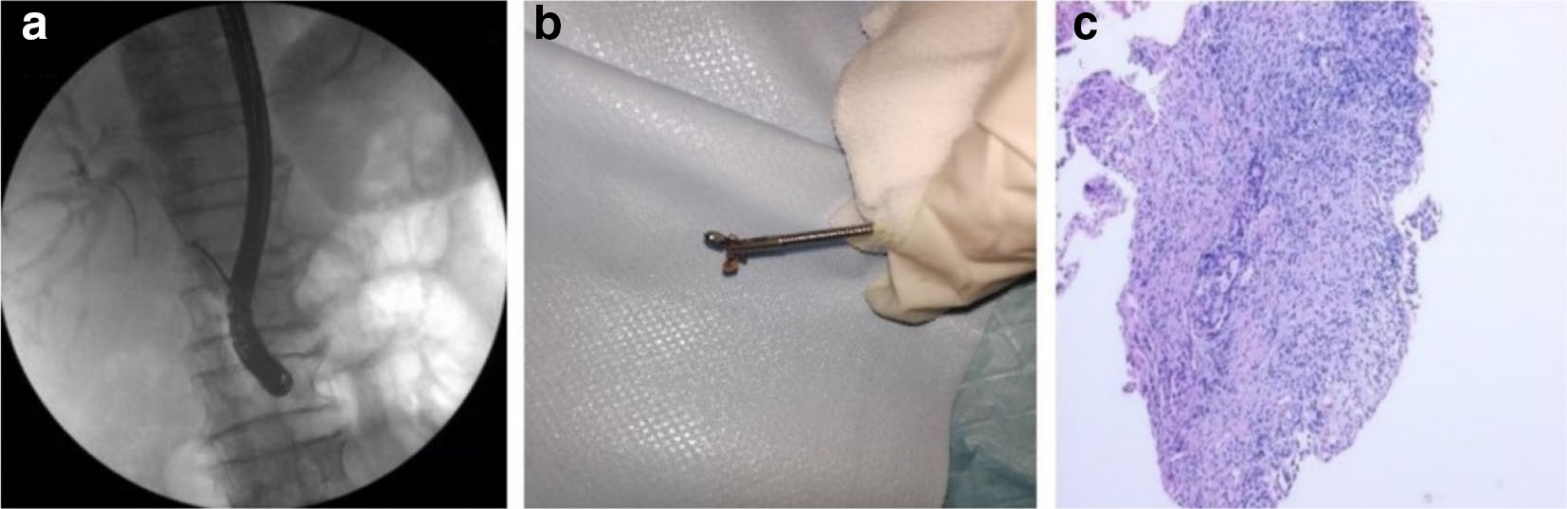
(**a**) Stenosis at the common hepatic duct and the upper segment of the common bile duct were approached and biopsied by the novel forceps under X-ray fluoroscopy. (**b**) The biopsy specimen obtained by elbow biopsy forceps. (**c**) Histopathological examination of the biopsy specimen showed adenocarcinoma.

Forceps were introduced into the bile duct without the assistance of a guide wire after ERCP was completed. Next, the stenosis sites were approached and biopsied by forceps under X-ray fluoroscopy (Supplementary Video 2). The tissue samples were fixed in 10% formalin for pathological evaluation (Fig. 3). All procedures were performed by an endoscopic physician with experience performing > 3000 ERCP procedures in our center. Histopathological examination was performed by two pathologists with 10 years of experience in bile duct histopathology.

### Follow-up

All patients with malignant lesions were treated surgically, and patients with benign lesions were followed up for at least six months by computed tomography or magnetic resonance imaging.

### Statistics

The measurement data are presented as the mean ± standard deviation (SD) or median (range), and the enumeration data are presented as percentages. Data were analyzed using 2019 Excel software.

## Results

### Information

Twenty-four patients (15 males and 9 females) were included in the present study. The average age of the patients was 68.3 ± 7.9 years old. Jaundice (66.7%) and abdominal pain (54.2%) were the most common clinical symptoms. The location of the stricture, size of the specimen, length of the stricture, number of biopsies and number of attempted biopsies are presented in Table 1.

**Table 1:** The patient characteristics.

Characteristics	
Number of patients, n	24
Sex (F:M)	9:15
Age, mean (SD), year	68.3 (7.9)
**Location of stricture, n (%)**	
Inferior	9 (37.5)
Middle	6 (25.0)
Superior	4 (16.7)
Hilar	5 (20.8)
Length of stricture, median (range), mm	13.5 (8–26)
**Clinical symptoms, n (%)**	
Jaundice	16 (66.7)
Abdominal pain	13 (54.2)
Anorexia	9 (37.5)
Fatigue	4 (16.7)
**Pathological diagnosis, n (%)**	
Malignant	17 (70.8)
Adenocarcinoma	6 (20.2)
Suspected adenocarcinoma	2 (16.7)
Severe dysplasia	9 (37.5)
Benign	7 (29.1)
Chronic inflammation	6 (25)
No tumor	1 (4.1)
Surgical treatment, n (%)	18 (75)
Following up, median (range), months	11 (7–20)
Number of attempt biopsies, median (range), n	5 (3–7)
Number of biopsies, median (range),n	2 (2–4)
The size of the specimen, mean (SD), mm	1.9 (0.4)

### Pathological diagnosis via biopsy

A total of 17 patients (70.8%) were diagnosed with malignancy base on biopsies. All of them underwent surgical treatment. Among them, nine cases (52.9%) were diagnosed as severe dysplasia, six cases (35.3%) were diagnosed as adenocarcinoma, and 2 cases (11.8%) were diagnosed as suspected adenocarcinoma. Chronic inflammation of the biliary duct wall was shown in the biopsy specimens of six patients (85.7%). No tumor was found in one patient (14.3%).

### Technical success and adverse events rates

All biopsy specimens could be used for histopathological examination, and the technical success rate was 100%. Two patients (8.3%) had mild postoperative pancreatitis and recovered shortly after treatment. No patients experienced biliary bleeding or perforation in our study (Table 2).

**Table 2:** Outcomes.

Technical success rate, n/N (%)	24/24 (100)
**Adverse events, n (%)**	
ERCP-related	2 (8.3)
Pancreatitis	2 (8.3)
Others	0 (0)
Forceps-related	0 (0)
Biliary bleeding	0 (0)
Biliary perforation	0 (0)
Pathological coincidence rate, n/N (%)	17/18 (94.4%)

### Pathological coincidence rates

The pathological coincidence rate was 94.4% (17/18) in the present study. The pathology of all surgical specimens was malignant (Table 2). Six patients with severe dysplasia based on biopsies were diagnosed with adenocarcinoma based on surgical specimens. All of them had jaundice and/or abdominal pain. Among these patients, four had biliary strictures located in the lower common bile duct, and the rest had strictures located in the middle common bile duct. The histopathological assessment of the surgical specimens and biopsy specimens of two patients showed severe dysplasia. Patients with adenocarcinoma and suspected adenocarcinoma on biopsies were diagnosed with adenocarcinoma based on surgical specimens. Pathological examination of the surgical specimen revealed mucinous adenocarcinoma in one patient with severe dysplasia on biopsy (Table 3).

**Table 3:** Pathological diagnosis based on biopsies and surgical specimens.

Patients	Diagnosis based on biopsies	Diagnosis based on surgical specimen
No.1	Adenocarcinoma	Adenocarcinoma, pancreatobiliary type
No.2	Adenocarcinoma	Adenocarcinoma, pancreatobiliary type
No.3	Suspected adenocarcinoma	Adenocarcinoma, intestinal type
No.4	Severe dysplasia	Adenocarcinoma, pancreatobiliary type
No.5	Severe dysplasia	Adenocarcinoma, pancreatobiliary type
No.6	Severe dysplasia	Adenocarcinoma, intestinal type
No.7	Adenocarcinoma	Adenocarcinoma, pancreatobiliary type
No.8	Severe dysplasia	Biliary intraepithelial neoplasia, high grade
No.9	Severe dysplasia	Adenocarcinoma, intestinal type
No.10	Adenocarcinoma	Adenocarcinoma, pancreatobiliary type
No.11	Severe dysplasia	Biliary intraepithelial neoplasia, high grade
No.12	Suspected adenocarcinoma	Adenocarcinoma, pancreatobiliary type
No.13	Severe dysplasia	Adenocarcinoma, pancreatobiliary type
No.14	Severe dysplasia	Adenocarcinoma, intestinal type
No.15	Severe dysplasia	Mucinous adenocarcinoma
No.16	Adenocarcinoma	Adenocarcinoma, pancreatobiliary type
No.17	Adenocarcinoma	Adenocarcinoma, intestinal type
No.18	Chronic inflammation	Adenocarcinoma, pancreatobiliary type

### Follow-up results

The median follow-up time was 11 (7–20) months in patients with benign strictures. No evidence of malignancy was found in the other six patients with benign lesions during the follow-up period. Three patients with benign lesions had jaundice, all of whom underwent endoscopic stent placement, and their jaundice subsided during the follow-up. The remaining three patients without jaundice had no malignancies on computed tomography and magnetic resonance imaging at the three- and six-month follow-up visits. One of the seven patients diagnosed with benign disease was found to have an advanced biliary stricture that was proven to be malignant during surgical treatment at the four-month follow-up visit after the ERCP procedure.

## Discussion

In this study, we successfully obtained adequate samples from 24 patients with biliary strictures. A total of 17 patients were diagnosed with malignant biliary stricture on biopsy. All of them underwent surgical treatment, and the pathological diagnosis of the surgical specimens confirmed malignancy. The findings of this study suggest that novel elbow forceps could obtain adequate samples from the bile duct.

In the present study, we assessed novel elbow biopsy forceps for sampling biliary strictures. The novel elbow biopsy forceps has an extremely flexible head; like a sphincterotome, the forceps could work with arbitrary angles and advance easily into the bile duct because of the special structure of the head. The elbow biopsy forceps have a wide clamp distance of 7.1 mm, which guarantees that we could obtain adequate specimens from the bile duct. In fact, all specimens we obtained could be used for histopathological examination.

In recent years, several technologies have been reported to be suitable for biliary tract sampling, such as endoscopic ultrasound-guided fine-needle aspiration (EUS-FNA) and percutaneous endobiliary forceps biopsy (PEFB)^11,12^. However, these technologies have some shortcomings. EUS-FNA has been used for the evaluation of pancreatic masses with high diagnostic accuracy, but it has some limitations in malignant biliary stricture cases outside of pancreatic masses^11^. PEFB is performed by means of the biliary pathway established by percutaneous transhepatic biliary drainage. The endobiliary forceps easily advances into hilar bile ducts during PEFB, but entering the common bile duct is difficult. PEFB is associated with more adverse reactions the use of biopsy forceps during ERCP^12^. All SpyBite specimens obtained by SpyGlass cholangioscopy can be used for histopathological examination. In their study, the mean size of the specimens was 1.1 ± 0.74 mm, which was smaller than that in our study^13^. Our study indicates that adequate specimens can be obtained by elbow biopsy forceps and used for diagnosis.

Accurate diagnosis of biliary strictures through biopsy is very important because biliary strictures have vastly different prognoses depending on their etiology. Biliary forceps biopsy or brush cytology is widely used during ERCP for the diagnosis of biliary strictures due to their easy of operation and inexpensive cost. Compared with biliary forceps biopsy, brush cytology is easier to perform but limited by its low sensitivity^14–16^. According to a meta-analysis, biliary biopsy was shown to have higher sensitivity than brush cytology^8^. Digital single-operator peroral cholangioscopy, which has outstanding maneuverability and excellent image quality, can be used for the diagnosis of biliary strictures^17,18^. A recent study reported that digital single-operator peroral cholangioscopy-guided biopsy has higher accuracy (87.1% vs 65.5%) in the diagnosis of indeterminate biliary strictures than conventional ERCP^19^. However, digital single-operator peroral cholangioscopy-guided biopsy is more expensive than ERCP-guided biopsy^18,20,21^. More often than not, patients tend to choose forceps biopsy procedures during ERCP in our department. According to the current European Society for Medical Oncology guidelines, ERCP-guided forceps biopsy can be used to obtain biliary tissue for histopathological examination^22^. However, obtaining specimens by conventional biopsy forceps is difficult because the quality of the sample cannot be guaranteed. The reason is that because of the straight head of the forceps, it is difficult for conventional biopsy forceps to advance into the bile duct because the approach is perpendicular to the direction of the bile duct. Furthermore, adjusting the direction of conventional biopsy forceps in the biliary tract is extremely difficult.

Controllable biopsy forceps were recently used to obtain specimens from the bile duct, and it had a control mechanism that could adjust the angle between the axis of the bile duct and the tip of the forceps. This study reported that the technical success rate was 99% (109/110)^23^. Controllable biopsy forceps can only be used in one direction because they have no rotational functionality. The bile duct wall can easily be approached on any side using the elbow biopsy forceps in our study because of its flexible, bent, and rotatable head.

One previous study reported that if malignancy is not confirmed within 6 months of the ERCP procedure, the strictures assessed can be considered benign^19^. In the present study, the median (range) follow-up for six patients with benign lesions on biopsy was 11 (7–20) months, and there was no deterioration. The pathological coincidence rate was 94.4% (17/18) in the study. One patient with benign lesions on biopsy had advanced biliary strictures that proved to be malignant during surgical treatment at the four-month follow-up. The reasons may be as follows: (1) we did not obtain malignant tissue because specifically targeting and performing biopsies of strictures are not precise processes under the guidance of X-ray; and (2) the lesion was not malignant when forceps biopsy was performed.

A recent Japanese study indicated that the incidence of post-ERCP pancreatitis was 10.2% (38/374), which was similar to the rate in our study (pancreatitis appeared in two patients)^24^. There was no evidence of biliary bleeding or perforation in our study, and it is unclear why elbow forceps insertion into the bile duct contributed to post-ERCP pancreatitis. The two patients who developed post-ERCP pancreatitis may have undergone endoscopic retrograde biliary stenting. This study shows that the biliary biopsy technique using elbow forceps is safe.

However, our study has several limitations. Our study took place in a single center, had a small sample size, did not include a control group and had a retrospective and nonrandomized design. We did not evaluate the sensitivity, specificity, and accuracy that resulted from using the elbow biopsy forceps for biliary strictures due to the small sample size. To verify our findings, a prospective, randomized multicenter trial study with long-term follow-up is required in the future.

In conclusion, the novel elbow biopsy forceps were safe and feasible for insertion into the bile duct and obtaining tissue from biliary strictures. The novel elbow biopsy forceps and the related biopsy procedures should be considered for indeterminate biliary strictures.

## Supplementary Information

Supplementary Video 1.

Supplementary Video 2.
